# High incidence of anterior cruciate ligament injuries in professional men's handball: Analysis of the ‘ACL registry in German Sports’ over seven consecutive seasons

**DOI:** 10.1002/jeo2.70132

**Published:** 2024-12-30

**Authors:** Tobias Resch, Werner Krutsch, Johannes Weber, Jonas Krueckel, Frederik Hartz, Volker Alt, Leonard Achenbach, Dominik Szymski

**Affiliations:** ^1^ Department of Trauma Surgery, Klinikum rechts der Isar Technical University of Munich Munich Germany; ^2^ Department of Trauma Surgery University Medical Center Regensburg Regensburg Germany; ^3^ FIFA Medical Center of Excellence University Medical Center Regensburg Regensburg Germany; ^4^ SportDocs Franken Nuremberg Germany; ^5^ Department of Orthopedics, König‐Ludwig‐Haus Julius‐Maximilians‐University Würzburg Würzburg Germany

**Keywords:** anterior cruciate ligament, handball, injury prevention, knee injury

## Abstract

**Purpose:**

The purpose of this study was to generate knowledge about the epidemiology and risk factors for anterior cruciate ligament (ACL) injuries in handball. It was hypothesised that the incidence of ACL injuries is high and comparable to other pivoting sports like football.

**Methods:**

This study is based on the prospective ‘ACL registry in German Sports’ implemented in the 2016–2017 season. Professional (first and second leagues) male handball players were analysed regarding the incidence and risk factors for ACL injuries. Injuries were registered according to the direct reports of the injured players to the study office and double‐checked via media analysis. After injury registration, the players received a standardised questionnaire. Data were analysed from the 2016–2017 to the 2022–2023 season.

**Results:**

A total of 84 ACL injuries were registered. 46.3% of ACL injuries were re‐ruptures. This represents a total incidence of 0.044 ACL injuries per 1000 h of exposure in training and matches per player over the study period. An increased incidence rate of 0.064/1000 h in the first league compared to 0.031/1000 h in the second league was reported (*p* ˂ 0.001). The mean number of injuries in the first division was 8.3, and in the second division, 6.6 ACL ruptures per season. Risk factors for ACL injury include previous knee injury and increased match exposure.

**Conclusion:**

The incidence of ACL injuries in professional handball is high and comparable to football with an increased risk in the first league compared to the second league in Germany. There was a high incidence of re‐injury. Other risk factors for ACL injuries include previous injuries to the affected knee in general as well as increased match exposure.

**Level of Evidence:**

Level II prospective cohort study.

**Abbreviations**
ACL: anterior cruciate ligament
BMI: body mass index
Max: maximum
MCL: medial collateral ligament
Min: minimum
SD: standard deviation
VBG: Verwaltungs‐Berufsgenossenschaft

## INTRODUCTION

Handball is a globally popular high‐intensity sport characterised by quick movements, sudden changes in direction, and physical contact between players [21]. With an average number of 1.9 injuries per player during one season, handball has the second‐highest injury rate in professional sports in Germany after football [15]. The International Olympic Committee ranks it among the Olympic sports with the highest injury rates [10, 15]. A recent systematic review on general injury incidence in handball involving all body regions per 1000 h of exposure found rates, of 7.8/1000 h in senior male players, 6.2/1000 h in female senior players and 6.9/1000 h in youth players [34]. The knee in general, and the anterior cruciate ligament (ACL) specifically, are among the most commonly affected areas, resulting in the longest periods of absence and highest costs [15, 17].

ACL injuries are particularly devastating for athletes because they often struggle to return to the same performance level and face potential long‐term risks such as osteoarthritis [38, 44]. Despite the high injury burden, there is a scarcity of literature focusing on ACL injuries in handball. The vast majority of studies report all injuries categorised by body region and general tissue without addressing the distinct injury entity such as ACL injuries. They often consist of small cohorts and include inhomogeneous data from amateur and professional levels, female, male and youth athletes [3, 6, 13, 16, 17, 19, 20, 22, 23, 35, 36], or report solely on single international tournaments [5, 41]. This complicates comparability between studies and the lack of knowledge about the epidemiology and risk factors of ACL injuries in handball makes implementing injury‐specific prevention measures challenging.

Several ACL registries have been established in Scandinavian countries, Luxembourg, the United Kingdom and the United States [33]. These registries offer detailed insights into the prevalence, basic epidemiology, diagnostic approaches and surgical considerations related to ACL injuries in the general population. While these ACL registries partially incorporate sports‐specific sub‐analyses for activities like handball, they lack prospective longitudinal data on ACL injuries among specific populations, such as professional handball players at different competition levels.

Therefore, a nationwide ‘ACL registry in German Sports’ (Kreuzbandregister im Deutschen Sport) was implemented at the beginning of the 2016–2017 season. It is based on the ‘ACL registry for German Football’, which started at the beginning of the 2014–2015 football season and was expanded to include professional handball, basketball and ice hockey [40]. The aim of the present study was to evaluate handball‐specific epidemiological data across different levels of play from this ACL registry with particular attention to risk factors for ACL injuries as a foundation for the implementation and improvement of preventive measures in the future. It was hypothesised that the incidence of ACL injuries in handball is high and comparable to other pivoting sports like football.

## MATERIALS AND METHODS

### Study population

This prospective cohort study is based on the nationwide ‘ACL registry in German Sports’ (Kreuzbandregister im Deutschen Sport) launched at the beginning of the 2016–2017 season. Informed consent was obtained from each patient. All professional male handball clubs in Germany (first and second league) participated in the study and reported any new ACL injuries of their players. From the 2016–2017 season onwards, the study included all male handball players with a new ACL injury who had actively played in the German men's professional leagues. Patients with both primary and secondary ACL injuries were included. Players with an ACL injury were excluded from prospective injury registration if they had not played at least one competitive match in one of the above‐mentioned leagues.

### Injury documentation and data collection

Before the study began in 2016 and the start of ACL injury registration, all handball clubs and their team physicians received an invitation to participate in the study and information on the aim, design and methods of ACL registration. All participants were annually reminded to take part in the study and to report new injuries to the study office.

After the occurrence of an ACL injury, a member of the team (the team physician, the injured player or a club official) reported the injury to the study office. Because of the size of the study population, the different medical settings of an inhomogeneous study population and the expected varying compliance of different players and clubs to the study guidelines, ACL injuries could not only be registered by players or club members but also by means of national media reports. Each week, national media reports on professional handball were systematically screened in various German online and print media. The use of online and print media for collecting data on severe injuries has been previously described and validated for professional handball [37]. In this ACL registry, such data were only used to double‐check the injury reports provided by the clubs. After the registration of a new ACL injury either through direct notification by the players and clubs or according to media reports, a standardised questionnaire in digital or paper form was sent to the injured player or the responsible team member. The injury reports for this study were adapted according to the commonly used injury report protocol in football established in 2006 by Fuller et al. [12] and according to previous epidemiological injury studies of this study group [37, 38, 39]. The protocol, which was sent directly after an injury, included anthropometric and sports‐specific data, any complaints before the injury, the player's past medical sports history, external and internal risk factors for ACL injury and detailed information about diagnostics, treatment and the occurrence of the ACL injury. Risk factors for ACL injury were divided into physical deficits (previous ACL injury or other knee injury to the same side, absence from handball for more than 4 weeks in the 6 months before ACL injury) and short‐term changes (move to a higher league, higher match exposure with less recovery time between matches, more than three matches in the 7 days prior to injury) to address both individual anatomical and physical issues as well as recent changes in exposure and load. All knee injuries with a time loss of at least two weeks were classified as prior knee injuries. If a club or player failed to provide the required injury data, the respective information was obtained from available media reports.

### Statistical analysis and data assessment

Injury incidence was calculated per 1000 h of exposure during matches and training according to Fuller et al. [12]. Match exposure was calculated by individual analysis of the official match schedules of each handball league. No data on the training exposure of individual players or teams were available in this registry. Training exposure was therefore estimated by means of literature reviews and the personal experience of several authors as former competitive handball players. Prevalence was defined as the proportion of injuries within the total population. Categorial variables were described as numbers and percentages of each category. Differences between groups were analysed with the chi‐square test or Fisher's exact test. Continuous variables were described as mean values with standard deviation. Intergroup comparison was done with the Student's *t* test or the Mann–Whitney *U* test for non‐parametric analyses, dependent on the normal distribution. *p‐*Values ˂ 0.05 were considered statistically significant. No sample size was calculated because the aim of the study was to recruit as many injured players as possible for the analysed seasons and the study design is prospective and explorative. The study office used the REDCap‐System, version 10.3.4 (Vanderbilt University), for data management. All analyses for this report were conducted using IBM SPSS Statistics, version 26.0 (IBM Corp.). The study design of the ‘ACL registry in German sports’ was approved by the local ethical committee (ID: 22‐2807‐101).

## RESULTS

**Figure 1:**
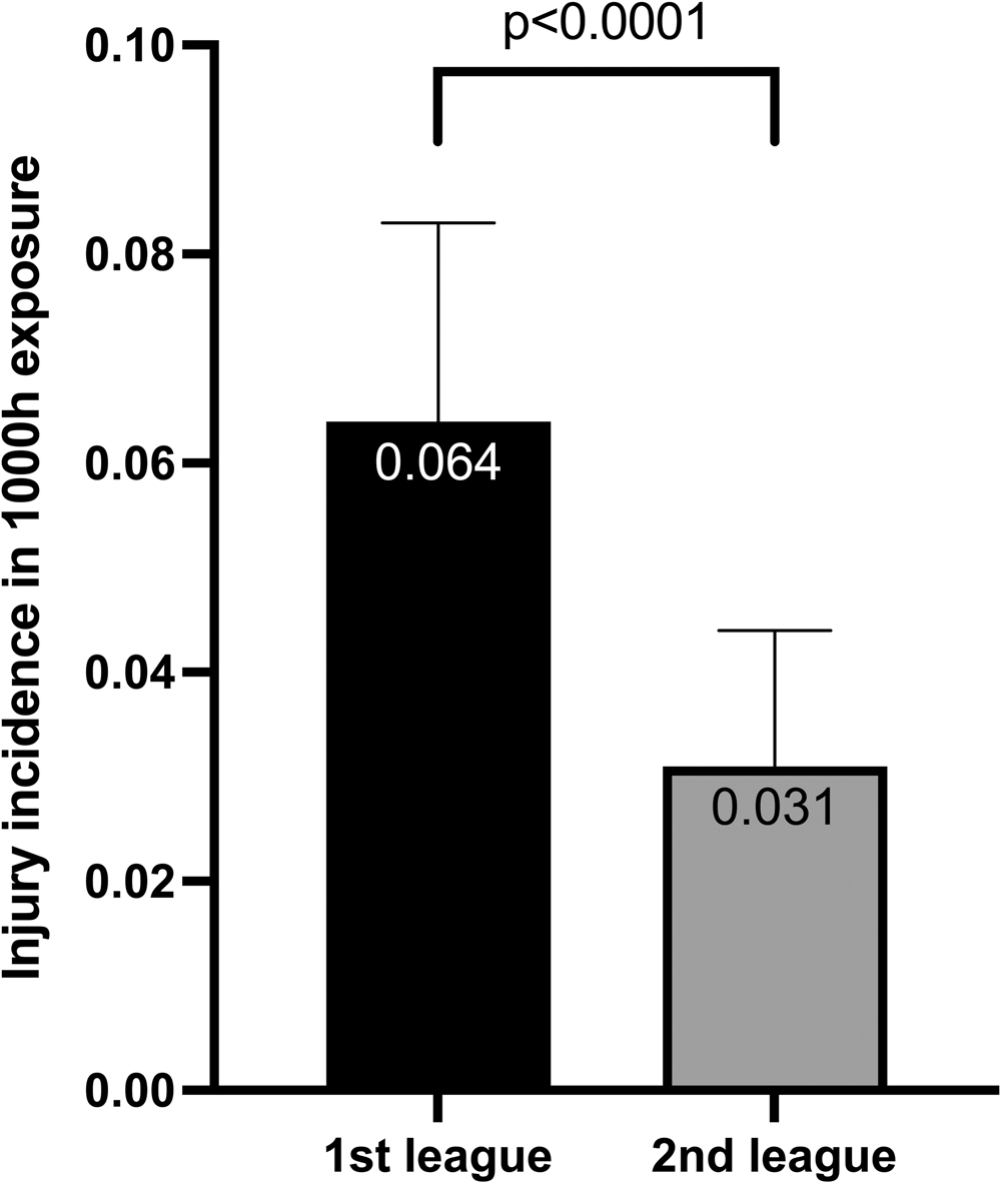
Cumulated ACL injury incidence in the first and second league in German team handball. ACL, anterior cruciate ligament.

**Figure 2:**
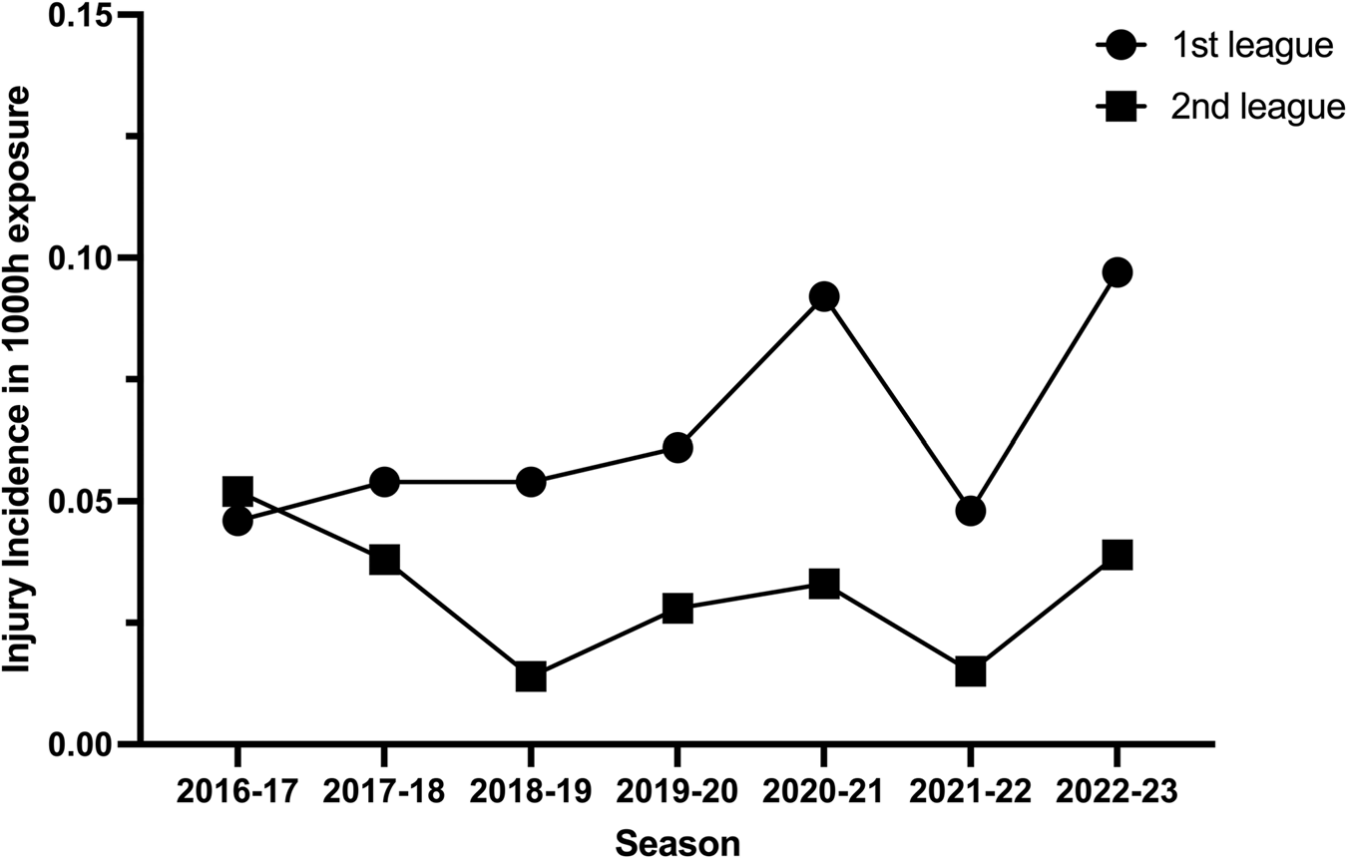
ACL injury incidence between 2016 and 2023 in the first and second German leagues in handball. ACL, anterior cruciate ligament.

Over the course of the investigated seven consecutive seasons, 5320 players (first league on average 374 athletes per season; second league 386 players per season) participated in professional handball in Germany, equally distributed across both leagues. Both groups showed similar anthropometric data (Table 1). A total of 84 ACL injuries were registered: 38 (45%) in the first league and 46 (55%) in the second league. The questionnaire was completed by 63 players (75%) with a sustained ACL injury. There was an estimated total incidence of 0.044 ACL injuries per 1000 h of exposure in training and matches per player over the study period. In the first division, on average, 8.3 injuries per season, and in the second division, 6.6 ACL ruptures per season were reported. A significantly higher incidence rate with 0.064/1000 h in the first league compared to 0.031/1000 h in the second league was demonstrated (*p* ˂ 0.001, Figure 1). The trajectory of injury incidence over the seasons in both leagues is depicted in Figure 2. On average, 8.3 injuries in the first league and 6.6 ruptures were reported in the second league per season. In 46.3% of the cases, the athlete experienced a re‐rupture of the ACL. This equals an estimated total incidence of 0.024 primary ACL injuries per 1000 h of exposure (0.035/1000 h in the first league vs. 0.017/1000 h in the second league). The risk factors for ACL injury are shown in Table 2, there was no significant difference between leagues. In 33.3% of the cases, the player had another injury to the affected knee joint before that resulted in an absence from sports for at least 2 weeks. Regarding the short‐term changes, 25% of the athletes reported increased match exposure before the injury with 20% having three games in the seven days preceding the ACL injury. In both leagues, the most frequent injury situation was landing (38.1%), followed by change of direction movements (17.3%) and stopping (12.9%) (*p* = 0.660, Table 3).

**Table 1:** Anthropometric data of the study population.

	Mean	SD	Min	Max
Age in years	26.1	9.5	17	45
Weight in kg	93.6	13.6	65	133
Height in cm	188.2	10.7	155	205
BMI in kg/m^2^	26.0	4.2	20.1	34.2
Experience in years	17.0	4.7	7	28

Abbreviations: BMI, body mass index; Max, maximum; Min, minimum; SD, standard deviation.

**Table 2:** Risk factors for ACL injury in the first and second handball leagues in Germany.

	1st league, *n* (%)	2nd league, *n* (%)	*p*
Physical deficits			
Previous ACL injury	15 (40.5)	16 (53.3)	0.293
Other previous knee injury (same side)	8 (36.4)	5 (29.4)	0.648
Absence from handball for more than 4 weeks in the 6 months before ACL injury	6 (22.2)	5 (17.2)	0.639
Short‐term changes			
Move to a higher league	9 (15.5)	10 (19.6)	0.073
Higher match exposure than usual	9 (27.3)	6 (22.2)	0.653
Three matches in the 7 days before ACL injury	9 (22.0)	5 (17.2)	0.627

Abbreviation: ACL, anterior cruciate ligament.

**Table 3:** ACL injury situation in the first and second handball leagues in Germany.

	1st league, *n* (%)	2nd league, *n* (%)
Acceleration	3 (9.1)	0 (0.0)
Running	2 (6.1)	3 (10.0)
Sprinting	1 (3.0)	2 (6.7)
Stopping	3 (9.1)	5 (16.7)
Change of direction	7 (21.2)	4 (13.3)
Jumping	1 (3.0)	1 (3.3)
Flight phase	0 (0.0)	1 (3.3)
Landing	13 (39.4)	11 (36.7)
Other situation	3 (9.1)	3 (10.0)

Abbreviation: ACL, anterior cruciate ligament.

## DISCUSSION

The most important finding of this study on ACL injuries in handball was the detection of a significantly increased injury incidence in the first league compared to the second league.

The cumulative incidence of ACL injuries was twice as high in the first league as in the second league. Two likely causes for this are the higher physical intensity, faster gameplay and greater mental pressure, as well as the increased match load due to additional international competitions with the clubs and matches with the national team [16, 36]. The resulting fatigue seems to play an important role, as significantly more injuries occur in the last 10 min of each half in games without an increase in foul play [17]. Therefore, prevention strategies should aim to monitor and manage training and match loads, identify and mitigate dangerous load peaks and enhance recovery periods [16].

In the first league, an increase in ACL injuries was observed over all seasons except for 2021/2022, which could be the result of reduced strain during the preceding COVID‐19 pandemic and the associated measures. A similar dip in injury incidence was also observed in the second league in the 2021/2022 season. In both leagues, the incidence increased again in the following year. The high incidence and burden of ACL injuries in football are well‐documented and extensively researched [24, 39, 43]. In contrast, while the risk of ACL injuries in handball appears to be comparable to that in football [39], it remains underrepresented in the scientific literature. In the late 1990s, Myklebust et al. followed 12 male elite Norwegian handball teams over the course of three seasons and found an ACL injury incidence of 0.06/1000 h for male athletes [28]. This number is similar to the cumulated injury incidence found in the first German handball league in the present study. Since the 1990s, the game speed, the number of matches and the level of athleticism increased, which places a significant demand on the musculoskeletal system [16]. For this reason, it could be assumed that the incidence of ACL injuries has increased in recent decades. Meanwhile, the professionalism of the medical care for the teams improved. From research, such as video analyses of ACL injuries, we now know more about the most common injury mechanisms and several promising prevention measures have been investigated [1, 2, 17, 26, 30, 31, 32].

In both leagues, most ACL injuries were non‐contact injuries occurring during landing, stopping and change of direction manoeuvres. These situations are frequently linked to intrinsic risk factors, including neuromuscular deficiencies and deficiencies in athletic performance or recovery and offer good targets for preventive measures [17]. Structured neuromuscular injury prevention programmes in handball have proven to be effective in reducing ACL injuries in prospective controlled intervention studies [1, 26, 31]. The training programmes used in these studies included various combinations of exercises aimed at improving balance, strength and agility [8, 9]. They also provided education on proper techniques for cutting manoeuvres and jumping/landing, awareness of potentially dangerous situations, as well as proprioceptive and plyometric exercises. A prerequisite for the effectiveness of these prevention programmes is sufficient compliance from the athletes [11, 14]. It has been shown that better compliance can be achieved among youth athletes compared to adults [31]. Therefore, implementing prevention programmes as early as possible can help raise awareness among young athletes, encouraging them to continue these training routines into adulthood. Achenbach et al. did not use any special equipment in their injury prevention programme, and the low financial investment required may facilitate the continuous implementation of such modules [1, 25]. Ultimately, these exercises need to be permanently integrated into the training routine. To achieve this, educating coaches and medical staff is essential, ideally facilitated by the respective handball associations [1].

This study showed that previous injuries to the affected knee joint are a relevant risk factor for suffering an ACL injury. In particular, a previous ACL injury was associated with a high rate of re‐injury. These factors have already been identified as risk factors for ACL injuries in football in a comparable study [39]. The high risk of a second ACL injury is particularly concerning, with reported rates exceeding 17% in the first five years following the initial ACL injury [42, 45]. The re‐rupture risk could not be determined in this study, as the number of players with a prior ACL reconstruction in the overall cohort was unknown. It is known that young male professional athletes are a high‐risk group for revision arthroscopies and ACL re‐ruptures [7, 46]. In this context, it is important to emphasise the significance of tertiary prevention, including the correction of neuromuscular deficits. An adequate rehabilitation period should be ensured and structured performance‐based return‐to‐sport testing should be conducted [29, 42]. However, treating surgeons can also influence the re‐rupture risk through their choice of surgical technique and graft selection [4, 46].

Short‐term increases in physical demands also seem to play a significant role in ACL injury development as 25% of injured players reported a higher match exposure than usual before injury. Once more, this underscores the importance of load management in ACL injury prevention.

This study demonstrates next to its benefits also several limitations. No data on the training exposure of individual players or teams were available in this registry. Therefore, the training exposure was estimated solely based on literature references and the authors' personal experience, which could skew the results. There was no differentiation between injury incidence in training and matches, and only male athletes were studied. However, a higher incidence of injuries during matches and a greater risk of knee and ACL injuries in women are well‐documented in the literature [13, 16, 18, 19, 27]. ACL injury data were gathered using two distinct registration methods: injury reports submitted by clubs or players and media screening conducted by the study office. These double‐checking methods ensure the maximum accuracy in identifying ACL injuries. Nevertheless, it should be noted that there is no guarantee that all injuries were captured. Due to compliance reasons, an injury protocol containing detailed information on the injury situation and intrinsic risk factors for an ACL injury was not available for every injured player. This carries the potential risk of selection bias, even though the majority of players did return the questionnaire. Furthermore, the number of examined risk factors was limited.

## CONCLUSION

The incidence of ACL injuries in professional handball is high with an increased risk in the first league compared to the second league in Germany. There was a high incidence of re‐injury. Other risk factors for ACL injuries include previous injuries to the affected knee in general as well as increased match exposure. This newly acquired knowledge about injury incidence and risk factors should be used to raise awareness of this devastating injury among athletes, coaches, medical staff and officials.

## AUTHOR CONTRIBUTIONS

Tobias Resch carried out the drafting of the article and participated in data analysis. Werner Krutsch, Volker Alt, Leonard Achenbach and Dominik Szymski conceived and coordinated the study. Werner Krutsch, Johannes Weber and Jonas Krueckel did the data collection. Frederik Hartz participated in data analysis and drafting of the article. Dominik Szymski and Leonard Achenbach contributed to data analysis, graphic representation and drafting of the article.

## CONFLICT OF INTEREST STATEMENT

The authors declare no conflicts of interest.

## ETHICS STATEMENT

The study design of the ‘ACL registry in German sports’ was approved by the Ethics Committee of the University of Regensburg (ID: 22‐2807‐101). Informed consent was obtained from all participants.

## Data Availability

All authors have read and approved the final manuscript.
